# Non-uniform space charge limited current injection into a nano contact solid

**DOI:** 10.1038/srep09173

**Published:** 2015-03-17

**Authors:** Y. B. Zhu, L. K. Ang

**Affiliations:** 1 Engineering Produce Development, Singapore University of Technology and Design, Singapore 639798

## Abstract

We have developed a two-dimensional (2D) non-uniform model to study the space charge limited (SCL) current injection into a trap-filled solid of nano-contact, such as organic materials and dielectrics. Assuming a solid of length *D* with a contact of width *W*, the enhancement over the well-known 1D uniform model is calculated as a function of *W/D* for different material properties, such as the dielectric constant (*ε*) and the trap distribution. The non-uniform current density profile due to edge effect is predicted. The findings reported here are different from the prior uniform 2D models, which are significant for small *W/D* when the size of the contact reaching nanometer scale, i.e. *W* = 50 nm for *D* = 1 μm. This model will be useful for the characterization of carrier mobility and properties of traps, which are critical to many novel devices (with small nano-contact) operating in the space charge limited condition reporting in novel device and its applications. Empirical formulas are given for future comparison with experimental results.

For high current transport in solids and organic materials, the space charge effect of the injected current is important, and it is known as the space charge limited (SCL) current injection. For a one-dimensional (1D) planar diode composed of a trap-free solid of length *D*, assuming the injection area is much larger than the material length, it is known as the 1D Mott-Gurney (MG) law^1^:
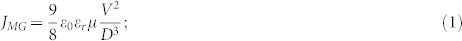
where *ε_r_* is the relative permittivity of the solid, *ε*_0_ is the permittivity of free space, *μ* is the electron mobility, and *V* is the applied voltage across the diode. For a trap-filled solid with exponentially distributed traps in energy, it is known as the trap-limited (TL) SCL current injection given by^2^,^3^:
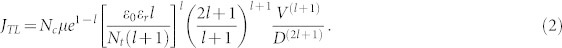
Here, *N_c_* is the effective density of states corresponding to the energy at the bottom of the conduction band, *N_t_* is the total trapped electron density, and *l* ≥ 1 is the ratio of distribution of traps to the free carriers. At *l* = 1, both equations give the well-known scaling of *V*^2^/*D*^3^. Note Eqs. (1) and (2) have been used widely to characterize the properties of solids such as mobility and trap distribution.

Recently, there are renew interests in SCL current transport in many novel devices involving either inorganic or organic materials, such as graphene oxide sheets^4^, light emitting diode^5^, organic device^6^, polymer transistor^7^, nanowire^8^,^9^,^10^, magneto-resistance^11^, photocurrent^12^, and nano-crystallites embedded silicon Schottky junction^13^. The effects on traps have also been extensively studied^14^,^15^,^16^,^17^,^18^,^19^ as the transport of SCL current as a function of voltage is very useful to characterize the properties of the traps and to indirectly measure the mobility of charge carrier. Other studies include temperature dependence transport behavior^19^,^20^,^21^, single electron model^22^, thermoelectric efficiency of nanowire in the SCL regime^23^ and the breaking of SCL in organic solar cells^24^.

In 2007, the 1D MG law has been extended to a 2D uniform model^25^ for an infinitely long emitting strip with width *W* by assuming a constant current density over the injection plane and drive the electric field on the middle point of that plane to zero, which indicates a geometrical enhancement of
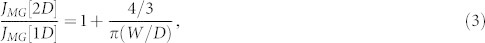
for *W/D* ≥ 1, and it agrees well with a device simulator (with error less than 5%). It was found that the enhancement factor decreases at higher *l* > 2 as compared to the trap-free case of *l* = 1^25^. Such geometrical enhancement effects had also been observed in nanowire but with a much smaller emitting area^8^,^10^ and much higher enhanced value. The crossover from the 1D to the 2D SCL current conduction behavior was also observed in single crystals^26^.

However, the 2D models^8^,^25^ have assumed uniform SCL current injection into the solid, which may be not valid due to the edge effect, especially when the typical size of the injecting area is smaller than the diode spacing. Note the condition to reach the space charge limited (SCL) current injection from a surface is to have sufficient high injected current density so that *all* electric fields on the surface can be driven to zero, which defines the *maximum* amount of steady-state current injection. From solving the Poisson equation, it is clear that it is impossible mathematically to have uniform profile of the injecting current density *J*, which is a constant along the emitting surface. Thus non-uniform profile of *J(x)* along the surface must be solved consistently especially for small emitting width *W* less than diode spacing *D*. At *W ≫ D*, our model will recover to the 1D SCL model shown in Eqs.(1) and (2). Physically, we can also see that to drive the higher electric field at the edge to be zero, higher current is required to be emitted near to the edge, so we have high localized space charge fields near to the edge.

Thus there is a need to construct a non-uniform model for SCL current injection into solid as prior uniform injection model may be no longer valid in many experiments in using nanoscale materials with small emitting size (in nm scale) with a diode (in micrometer scale). For simplicity, we will focus on a 2D non-uniform model having a finite emitting width of *W* (see Fig. 1 below).

Note the classical MG law [Eq. 1] is based on a 1D model by assuming the size of the injection width is infinitely large (*W ≫* *D*). Thus the calculated space charge limited (SCL) density *J* is only a function of *V* and *D*, independent of *W*. In this paper, we consider finite emitting size, and develop a 2D non-uniform model to include the dependence of *W* or ratio of *W/D*. This indicates that such a 2D model will be required to solve two-dimensional Poisson equation consistently with appropriate boundary conditions as shown in Eqs. 4 and 5. It is important to note that non-uniformity is important for *W* < *D*, and the prior 2D uniform models^25^,^27^ are only approximately correctly for finite *W* > *D*.

For charge injection into a solid, the property of contact is important – it can be either an Ohmic contact or Schottky contact. However when the charge injection is at high current regime as studied here, the type of contact is less critical when the SCL condition (high current) is reached. For example, the 2D uniform MG law^25^ for an Ohmic contact has been verified with Schottky contact^27^ and it is confirmed that both models gives the same enhancement factor. For simplicity, we have assumed Ohmic contact in this paper.

The approach presented in this paper can be extended to complicated 3D geometries such as nanowires and nanorods, which will serve for future works and comparison with prior model such as Ref. 8. Note we have not yet developed a 3D (or protrusive) and non-uniform model to account for the emission from nanorods like Ref. 8. If we ignore the sharpness, and simply assume a “flat” cylindrical emitting shape of a finite radius *r*, our calculated results from this 2D “flat” non-uniform model provide lower values as compared to Ref. 8. We believe that it is due to the sharpness of the nanorod which has been ignored completely. For completeness, the high electric field enhancement near to the tip of the nanorod should be included. Since the space charge effect is trying to drive the higher electric field to become zero, thus we speculate higher SCL current density will be obtained if the sharpness is included in the model.

## Results

### Non-uniform injection

**Figure 1:**
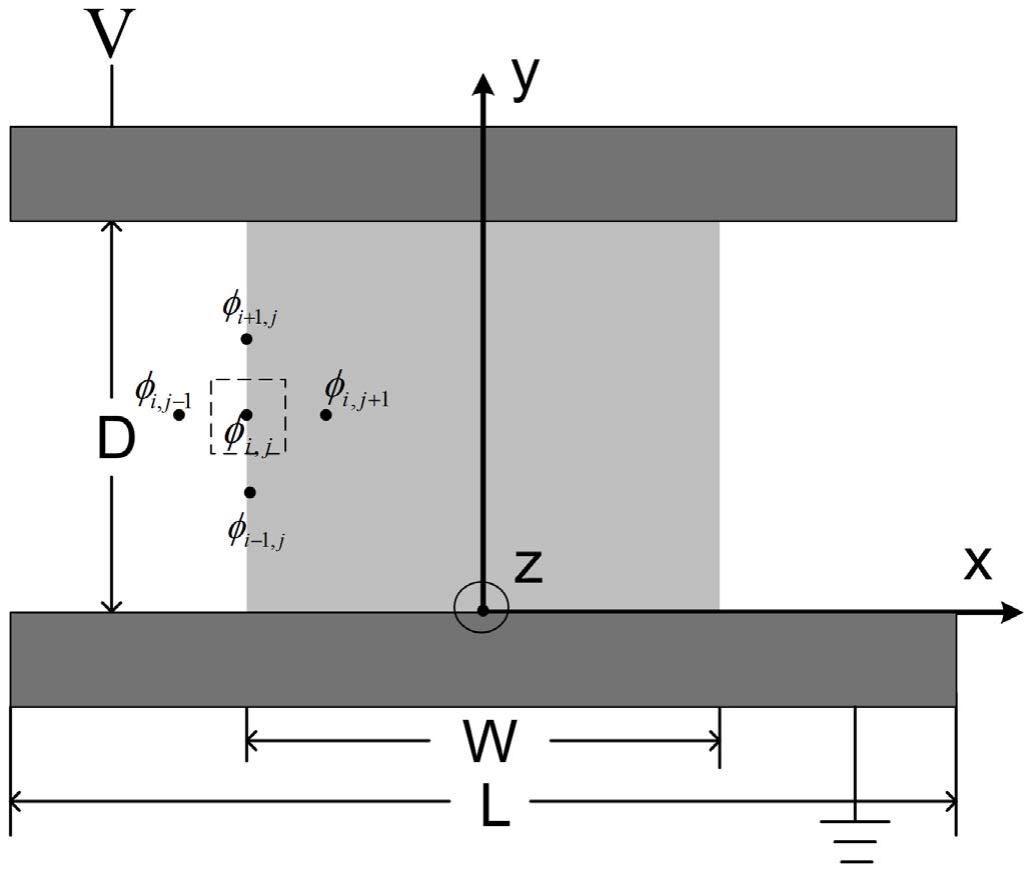
Schematic diagram of SCL electron injection into a solid inside a planar diode with a spacing *D*. The solid has a finite width of *W* and is infinitely long in *x* direction.

As shown in Fig. 1, we first consider a trap-free solid of length *D* and width *W*, which is sandwiched between two metallic electrodes with a biased voltage of *V*. The solid is assumed to be infinite long in the *z*-direction. The size of both electrodes is *L*, which is assumed to be much larger than *W*. The electrons are injected from the lower electrode (*y* = 0 and −*W*/2 ≤ *x* ≤ *W*/2) into the solid. The interface is assumed to be an Ohmic contact and the effect of Shottky barrier on the SCL current injection^27^ is ignored completely. The drift component of the current density is given by *J*(*x*) = *ρ*(*x*,*y*)*μE_y_*(*x*,*y*), where *ρ*(*x*,*y*) is the charge density and *E_y_*(*x*,*y*) is the electric field in the *y*-direction. On substituting from the charge density *ρ*(*x*,*y*) into the Poisson equation, we obtain the following normalized differential equations:
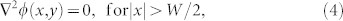

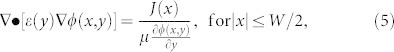
which can be solved numerically with the following boundary conditions:
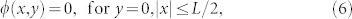

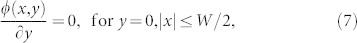

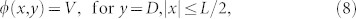

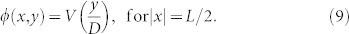
In our model, the non-uniform injection of current density is characterized by *J*(*x*) in Eq. (5). Its dependence on *x* is required to be determined numerically subjected to the boundary conditions. Here, the SCL injection is defined by having the electric field equals to zero at all the injecting points at the lower electrode as shown in Eq. (7). Note it is not trivial to solve Eqs. (4)–(9) due to this unknown *J*(*x*) parameter. From the analysis, we find that only two of the boundary conditions in Eqs. (6), (7) and (8) can be satisfied if a constant *J* has been assumed in solving Eqs. (4) and (5), which explains mathematically why a non-uniform model is required. Thus, one can image that the current density would have a non-uniform profile like having a higher value near to the edge in order to fulfill the boundary conditions.

**Figure 2:**
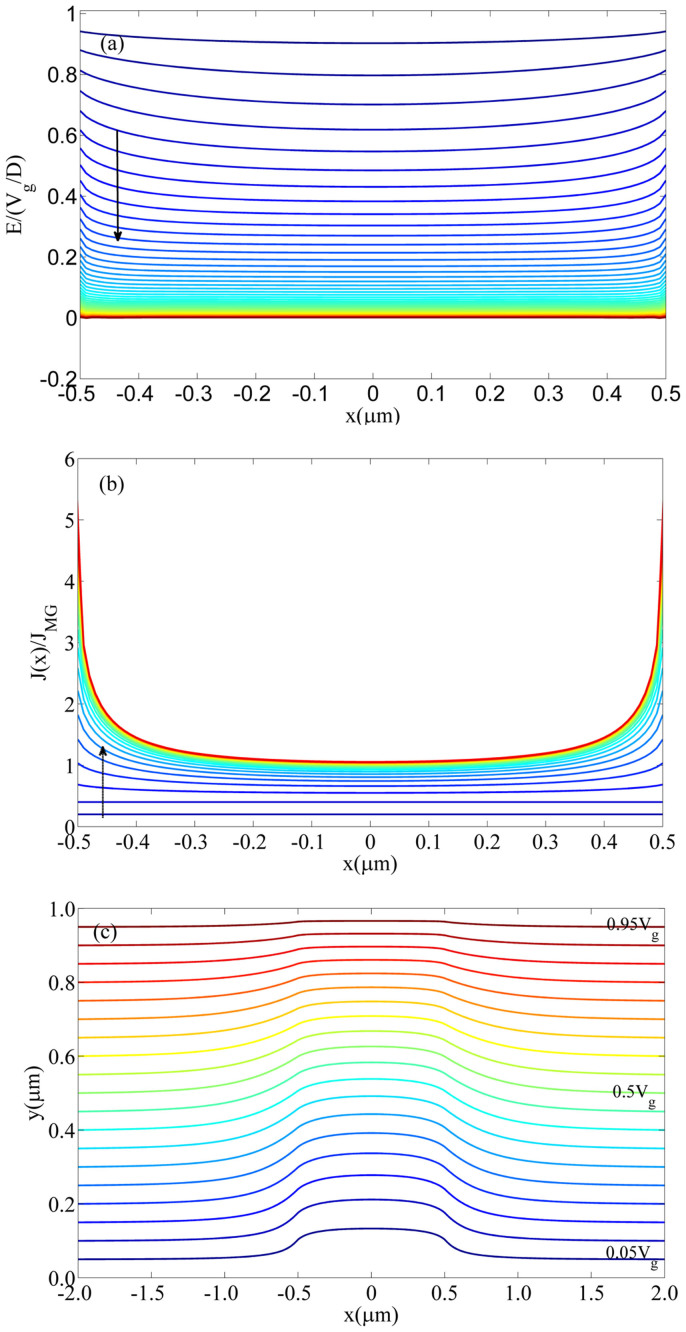
The numerical evolution (indicated by the arrows) of (a) the normalized electric field at the injecting surface, (b) the normalized current density, and (c) the equal potential curve distribution under non-uniform SCL injection at *W/D* = 1 and *D* = 1 μm. Here we have fixed *L/D* = 4.

**Figure 3:**
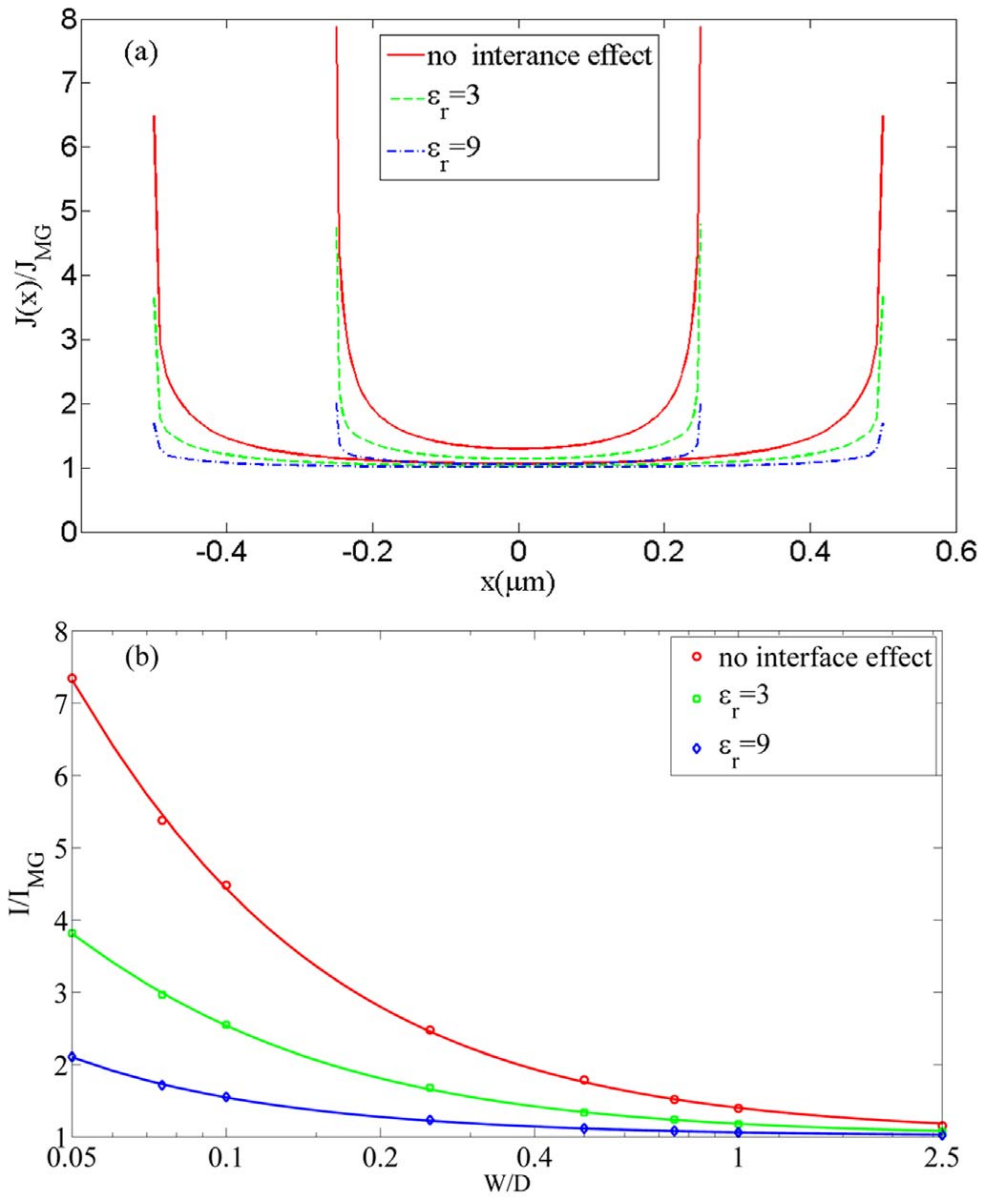
(a) The non-uniform profile of the normalized SCL current density *J* for a trap-free solid at *W/D* = 0.5 and 1 (fixed *D* = 1 μm) for PPV films (*ε_r_* = 3) and GaN (*ε_r_* = 9). (b) The geometrical enhancement of the 2D non-uniform MG law over the 1D MG law as a function of *W/D* and *D* = 1 μm. The cases of no interface effect mean we do not consider the change of the dielectric constant change at vacuum-solid interface and simply use the dielectric constant of the material.

In Fig. 2, we show the numerical evolution (indicated by the dashed arrow) of the normalized electric field, and its corresponding current density at *W/D* = 1 for *D* = 1 μm. As the injection current density increase, the electric field on cathode surface approach zero (see Methods). From the figure, we clearly see a wing-like profile, which shows a larger current enhancement of *J* near to the edge. We repeat the calculation at different *W/D* = 0.5 and 1, which are plotted in Fig. 3(a). For each *W/D* case, we calculate the effects due to the dielectric constant *ε_r_* at the interface, which are *ε_r_* = 3 (dashed lines) for PPV films^28^, *ε_r_* = 9 (dashed-dotted lines) for GaN^8^ and without the interface effect (solid lines). Here the cases without the interface effect show that the enhancement is *ε_r_* independent. It is clear that the enhancement near to the edge reduces at high *ε_r_* due the reduction of the effective electric field at the interface given by *V*/(*Dε_r_*).

By using *J*(*x*), the total SCL current (*I*) is calculated by integrating *J*(*x*) over the width of the injecting surface. The results normalized to the 1D MG law [see Eq. (1)], *I/I_MG_* are plotted in Fig. 3(b) in the range of 0.05 ≤ *W/D* ≤ 2.5. Here we have *W* = 50 nm to 2.5 μm for *D* = 1 μm. The results may be fitted into an empirical scaling of
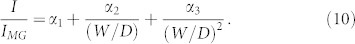
Here, the three constants (α_1_, α_2_, α_3_) are, respectively, (1.0384, 0.3647, −0.0025), (1.016, 0.1651, −0.0013) and (1.0102, 0.0524, 0.0001) for no interface effect (circle), ε_r_ = 3 (square), and ε_r_ = 9 (diamond).

**Figure 4:**
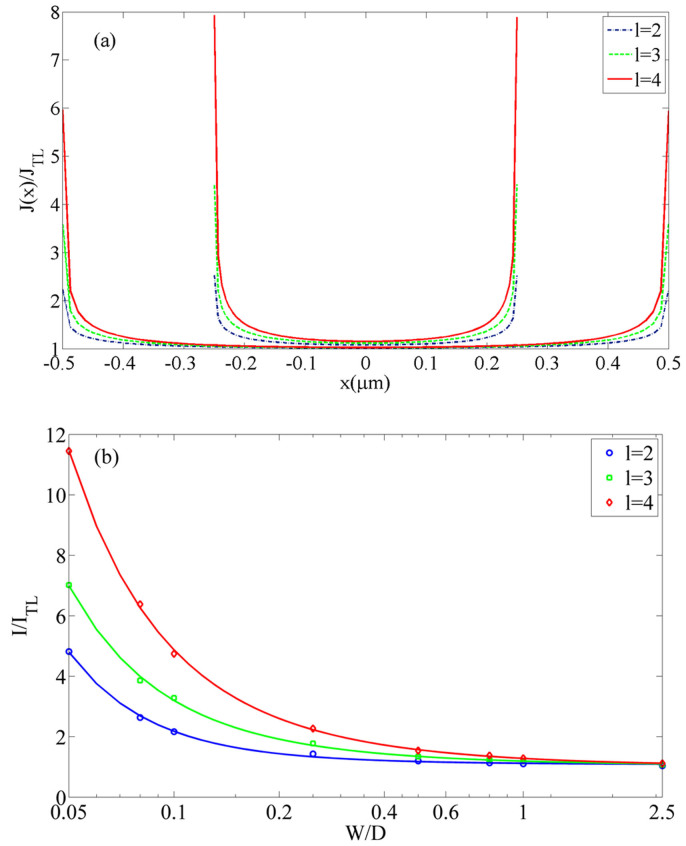
(a) The non-uniform profile of the normalized SCL current density *J* for a trap-filled solid like Si (*ε_r_* = 11.8) with different *l* = 2, 3, 4 at *W/D* = 0.5 and 1 for *D* = 1 μm, (b) The geometrical enhancement of the 2D non-uniform trap-limited current over the 1D model as a function of *W/D* and *D* = 1 μm.

In our simulation (see Fig. 3b and 4b), we fixed *D* = 1 μm, and *W* is varied from 50 nm to 2.5 μm (*W/D* = 0.05 to 2.5), so the contact size is less than 100 nm, and it is at nanometer scale. There is no problem to even smaller value of *W*, but it will take intense computation time. We have done a case down to *W* = 5 nm (*W/D* = 0.005) at *ε_r_* = 3 [it requires long simulation time for convergence], the calculated value is *I/I_MG_* = 144.

### Effects of Traps in solid

To include the effects of traps in a solid, we consider the energy state of the traps solid is described by an exponential function *N*(*E*) = (*N_t_*/*kT_c_*)exp[(*E* − *E_c_*)/*kT_c_*]. Based on Eq. (2), Eq. (5) becomes
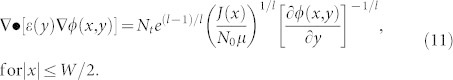
Using the same numerical approach [see Methods], we then calculate the 2D non-uniform trap-limited current density as a function *W/D* for different *l* and *ε_r_*.

In Fig. 4(a), take Si as an example (*ε_r_* = 11.8), we show the profile of *J*(*x*) over *J_TL_* at *l* = 2, 3, 4 for various *W/D* = 0.5 and 1 at *D* = 1 μm. One can see that the current density also increases at the edge, and the enhancement increases with higher *l*. Compared to *l* = 1 case (similar to the trap-free case as shown in Fig. 3a), enhancement for *l* = 2 and *l* = 3 is more significant.

Similarly the total trapped limited current [in terms of Eq. (2)] is shown in Fig. 4(b). Using the same scaling of Eq. (10), we also obtain the fitting constants, which are respectively, (1.0817, 0.0335, 0.0076), (1.0537, 0.1319, 0.0082), (1.0218, 0.2478, 0.0137) for *l* = 2, 3 and 4. It is interesting to note that, the geometrical enhancement is more important for trap-limited current for non-uniform model (as present here) as compare the 2D uniform model presented before Ref. 25. For the uniform model, it was found that the geometrical enhancement decreases from *l* = 1 to *l* = 2 and 3 (see Fig. 3 in Ref. 25). We can confirm this finding by having uniform ejection in our model, which recover the prior findings as reported in Ref. 25.

## Discussion

In this work, we have presented a 2D *non-uniform* SCL current injection into a solid (of width *W*) with and without traps. Empirical scaling has been provided for the geometrical enhancement factor as function of *W/D* for different dielectric constants and trap distribution. For a fixed *W/D*, it is found that the enhancement (over the 1D model) is higher for a solid with smaller dielectric constant and with larger energy spread in the trap distribution. Compared to prior uniform model, we observed higher local current density near to the edge. The model will be useful for the characterization of material's properties like mobility and trap distribution, especially for a solid (with a nano-contact size) operating at the SCL regime.

From recent experimental results^8^, it was suggested that the threshold voltage to reach the SCL limited current will be lower for nanowires. Thus the non-uniform model presented in this paper should be useful to characterize the high current SCL transport in nanowires at the regime when SCL conditions are reached at all surfaces. The non-uniformity of the SCL current injection presented here will be relevant to any injecting surface, especially for nano-contact areas for organic materials and others^29^. The different dependence on the trap distribution may be useful to determine if uniform or non-uniform injection is favourable in a particular solid. As an example, this model would be useful for studying the SCL electron injection into quantum dot LED which has very large value of *l* and small contact size^30^.

Using our non-uniform model presented here, there are 2 methods to check if non-uniformity is important from the experimental results (like thin-film like contact). Our model (see Fig. 3b) has indicated the enhancement decreases with higher dielectric constant, such dependence (due to the discontinuity of dielectric constant at the edge between the solid and free space) is absent from the prior uniform model, which has overestimated the enhancement (see the red line in Fig. 3b). To compare with uniform model [like Ref. 25] assuming *W/D* = 1, which gives an enhancement of 1.43 (independent of ε_r_) and it is higher than our results namely 1.4 (no discontinuity), 1.18 (ε_r_ = 3) and 1.06 (ε_r_ = 9).

The second method is to measure the enhancement for a solid with different trap distribution at *l* = 2, 3 and 4. Our model [see Fig. 4b] shows the enhancement decreases with smaller *l*, while the uniform model [see Fig. 3 in Ref. 25] showing the different trends. If this finding can be verified experimentally, the model would be useful to characterize trap-dominated organic based materials for which non-uniformity issue may be relatively more important.

## Methods

In our calculation, we first assume a specified *J* in an inner iteration step, and solve Eqs. (4) and (5) numerically for *ϕ*(*x*,*y*). Here, Eq. (5) is rewritten as
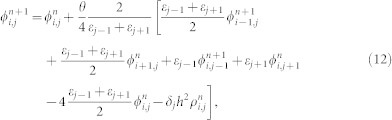
where *ε* is the dielectric constant, *h* is the grid size, *n* + 1 and *n* is, respectively, the *(n + 1)*-th and *n*-th iterative step. Here, *θ* (a value between 1 and 2), is a relaxation parameter used to accelerate the iteration, *δ_j_* is 0, 1 and 0.5, respectively for the grid point in vacuum, dielectric and vacuum dielectric interface. The charge distribution is given by 
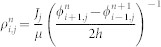
. Note Eq. (12) is solved by using a nonlinear poison solver, which gives the potential and charge distribution self consistently.

From the numerically solved potential distribution *ϕ*(*x*,*y*), one can only calculate the electric field half a numerical grid from the injecting electrode. In the SCL regime, the electric field varies rapidly near to the electrode, which makes the calculation of the electrode surface electric field difficult. The first numerical grid plays an important role in determine the current density. In order to obtain the cathode surface electric field, we use the following extrapolating function,
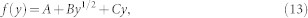
where *A* is defined as the normal component of the electric field. By using Eq. (13) and *ϕ*(*x*,*y*), at the 3 nearest half-grid points (to the injecting electrode), we can numerically solve for *A, B* and *C*, and thus obtain the surface electric field through the value of *A*. For the trap-limited case, due to the different scaling for electric field at different *l*, the extrapolating function to obtain the electric filed on cathode surface becomes
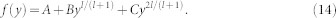
In order to push the electric field to zero, we have another outer iteration process using the scant method to update the spatial profile of *J*(*x*) consistently^31^. Firstly, we start with two uniform current densities which is a fraction of the 1D MG law. For example, we normally use *J*_1_ = 0.2*J_MG_* and *J*_2_ = 0.4*J_MG_*. We then solve the restricted nonlinear Poisson equation by considering only the Dirichlet boundary condition to obtain *ϕ*(*x*,*y*) for a given current density *J*(*x*) (in the inner loop). The corresponding electrical fields on the surface are then numerically calculated as *E*_1_ and *E*_2_, respectively. By using a secant method, we obtain an intermediate current density J_*_ given by
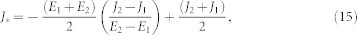
and the new current density is updated by *J_new_* = 0.1J_*_ + 0.9*J_old_*. This numerical process will continue until the convergence in the calculated electric field is better than 0.01%.

## Author Contributions

Y.B.Z. and L.K. A conceived the idea. Y.B.Z. developed the model and the numerical code. Both people discussed the results and wrote the paper.
